# Research on Vehicle Pose Detection Method Based on a Roadside Unit

**DOI:** 10.3390/s24144725

**Published:** 2024-07-21

**Authors:** Juan Ni, Xiangcun Kong, Bingchen Yan, Shuzhe Si, Shuyue Shi, Dong Guo, Pengwei Wang, Lei Wang, Yi Xu

**Affiliations:** 1 School of Transportation and Vehicle Engineering, Shandong University of Technology, Zibo 255000, China; 22502060003@stumail.sdut.edu.cn (J.N.); 22502060006@stumail.sdut.edu.cn (X.K.); 22502060007@stumail.sdut.edu.cn (S.S.); guodong@sdut.edu.cn (D.G.); wangpw@sdut.edu.cn (P.W.); 2 Zibo Environmental Pollution Control and Prevention Center, Zibo 255000, China; jdcpqwrjck@zb.shandong.cn; 3 Cestera Motor Co., Ltd., Zibo 255000, China; sishuzhe@suntae.cn (S.S.); wanglei@suntae.cn (L.W.); 4 Realepo Technology Co., Ltd., Zibo 255000, China; 5 Qingte Group Co., Ltd., Qingdao 266106, China

**Keywords:** RSU, UDP, vehicle detection, pose detection

## Abstract

Vehicle pose detection plays a vital role in modern automotive technology, which can improve driving safety, enhance vehicle stability and provide important support for the development of autonomous driving technology. The current pose estimation methods have the problems of accumulation errors, large algorithm computing power, and expensive cost, so they cannot be widely used in intelligent connected vehicles. This paper proposes a vehicle pose detection method based on an RSU (Roadside Unit). First, the on-board GPS performs the positioning of the target vehicle and transmits the positioning information to the RSU through the UDP (User Data Protocol). Next, the RSU transmits a forward command to the OBU (On-board Unit) through the UDP. The OBU sends the command to the ECU (Electronic Control Unit) to control the vehicle forward. Then, the RSU detects and tracks the vehicle. The RSU takes pictures of two images before and after the movement and obtains the coordinates of the four angle points and the center point by image processing. The vehicle heading direction is determined by the moving direction of the center point of the front and rear two images. Finally, the RSU captures the vehicle images in real time, performs the process of tracking, rectangular fitting and pose calculation to obtain the pose information and transmits the information to the OBU to complete the whole process of vehicle pose detection and information transmission. Experiments show that the method can realize accurate and efficient detection of vehicle pose, meet the real-time requirements of vehicle pose detection, and can be widely used in intelligent vehicles.

## 1. Introduction

As a key vehicle perception technology, vehicle pose detection can accurately identify and understand the movement state of the vehicle, so that the driver or the intelligent driving vehicle can make more accurate and safe driving decisions. In the field of intelligent driving, vehicle pose detection can also provide key data support for automatic driving systems, and improve the perception of road conditions and obstacles, so as to better cope with the complex traffic environment. Therefore, the research on vehicle pose detection is necessary and meaningful. The camera has the advantages of easy installation, strong adaptability to the environment and low cost, so it has an important application value in vehicle pose detection. At the same time, with the rapid development of V2X (Vehicle to Everything) communication technology, RSU (Roadside Unit) plays an increasingly important role in intelligent transportation systems. Through its positional advantages, extensive coverage, and efficient data transmission capabilities, RSU provides strong support for realizing intelligent driving and intelligent transportation systems. The vehicle pose detection method based on the RSU can provide reliable pose information in real time due to the simplicity of its solution method and the high camera frame rate. With the advantages of low environmental requirements, strong adaptability, and low cost, this method can compensate for the disadvantages of easily losing GPS signals, the high cost of radar, and sensor drift, making it widely applicable.

This paper proposes a vehicle pose detection method based on the RSU, which can obtain vehicle pose information in real time and with high precision, laying a foundation for the vehicle in stability control, driving safety, and path planning. The remaining structure of the paper is as follows. The second section discusses the current vehicle pose detection methods. The third section details the rectangular fitting method, the vehicle pose calculation method, and the vehicle heading direction detection method. The fourth section shows the training results of target detection and the experiment results of this method in the real vehicle scenarios. In the fifth section, the proposed method is summarized and the subsequent research is also presented.

## 2. Related Works

Accurate vehicle pose information is the key component of autonomous driving systems. Scholars, both domestically and internationally, have conducted extensive research on vehicle pose detection methods. At present, the mainstream vehicle pose detection methods are the camera-based methods, the lidar-based methods, and the multi-sensor combination algorithms.

Because of the ability of cameras to capture rich image information and the increasing maturity of deep learning algorithms, camera-based vehicle pose detection methods are widely used. The camera-based methods can automatically extract and learn abstract features from the data, which have strong adaptability to the vehicle pose changes under different angles and light conditions. Xu et al. [1] integrated deep learning with enhanced digital maps to accurately estimate vehicle heading angles. Huang et al. [2] presented a lightweight deep learning framework for effective prediction performance in unobscured vehicle scenarios. Gupta et al. [3] introduced an efficient vehicle pose estimation architecture based on multi-scale deep learning for precise estimation with manageable network complexity. Zhao et al. [4] proposed a deep learning-based FPN method for monocular camera-based vehicle pose estimation to improve speed and accuracy. Lyu et al. [5] used the vehicle 3D priors to estimate the 6D pose and improved the detection accuracy and speed. The camera-based methods rely on large amounts of labeled data and have limited generalization ability in specific scenarios, while having a high demand for computational resources.

The lidar-based methods can provide a high precision distance measurement and can accurately capture the details of the vehicle surrounding environment, thus enabling 3D positioning and attitude detection of the vehicle. Wang et al. [6] introduced a lidar-based method for estimating the pose and velocity of the ego vehicle and surrounding moving obstacles simultaneously. Zhao et al. [7] presented a real-time tracking algorithm using L-Shape fitting, enhancing performance and accuracy. Yang et al. [8] proposed a vehicle pose estimation method based on edge distance for boundary rectangle-based pose estimation. Gu et al. [9] developed a registration algorithm with road information constraints, demonstrating accuracy and effectiveness in practical use. The lidar-based vehicle pose detection methods provide superior robustness in complex environments by exploiting 3D geometric information. However, the lidar-based methods may face limitations in some applications due to their uneven density, high processing complexity, and the dependence on precise registration and localization.

The multi-sensor combination algorithms have a wide range of applications, especially playing a key role in environmental perception as well as in navigation and positioning. By integrating data, more comprehensive, accurate, and real-time information acquisition from different sensors is enabled. Wu et al. [10] added a GPS heading angle to the measurement vector, and established its error model, which effectively improved the accuracy of the GPS heading angle. Balazadegan Sarvrood et al. [11] proposed a method that combined visual odometry, light detection and ranging odometry, and a simplified inertial measurement unit. This method achieved an accurate estimation of pose information in urban areas with surrounding buildings. Xu et al. [12] proposed a new multi-information fusion vehicle pose estimation method to achieve precise vehicle pose estimation even during GNSS interruptions. Yang et al. [13] proposed a method which used 4D radar and a camera to robustly estimate vehicle pose to use the complementary characteristics of each sensor. The method showed excellent performance and enhanced robustness. An et al. [14] fused inertial and multi-antenna GNSS measurements to provide higher precision for positioning and attitude estimates. To address the unique demands of safety-critical applications in challenging observation conditions, Li et al. [15] introduced an innovative tightly integrated RTK (Real-Time Kinematic)/INS (Inertial Navigation System) algorithm to ensure the continuous delivery of precise and dependable positioning outcomes. Bersani et al. [16] presented an innovative integrated algorithm that provided an accurate estimation of the vehicle pose for the panning algorithm in multiple challenging scenarios. To identify the pose accurately, CNS (Celestial Navigation System) is commonly combined into the INS/GNSS integration, leading to an INS/GNSS/CNS integrated navigation system [17,18,19,20,21]. The multi-sensor combination algorithms have the advantages of improving accuracy, enhancing robustness and adapting to multiple scenarios, but also face challenges of complexity, cost, and energy consumption.

Based on the above research, the current camera-based vehicle pose detection methods have limited generalization ability in specific scenes and have a high demand for computing resources; the lidar-based methods have a high cost, processing complexity, and severe dependence on accurate registration and positioning; and the multi-sensor combination algorithms have high complexity and are not widely used. In this paper, RSU is used to realize vehicle pose detection, UDP communication is used to realize information transmission between OBU and RSU, and the whole detection process is completed by controlling vehicle forward, vehicle heading direction detection, target detection and tracking, vehicle rectangle fitting, and pose calculation. This method solves the problems of high pose detection power, high cost, and weak real-time performance. With the development of Internet of Vehicles technology, this method has great practical application significance.

## 3. Method 

**Figure 1:**
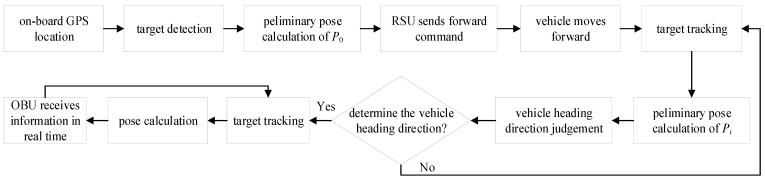
Flow chart of vehicle pose detection method based on the RSU.

The vehicle pose detection method based on the RSU can detect the vehicle pose without any prior information and meet the real-time requirements in the actual process of vehicle driving. Figure 1 shows the specific flow of the vehicle pose detection method based on the RSU.

(1) In order to accurately identify the target vehicle in complex road conditions, the initial position information of the target vehicle is obtained by using the on-board GPS, and the information is transmitted to the RSU through UDP in the OBU.
(2) After receiving the position information of the on-board GPS, the RSU camera takes pictures of the vehicle on the road and performs the target detection. Since the GPS position information of the vehicle has been obtained, the target vehicle can be extracted from the target detection.
(3) Preliminary pose calculation is performed on the initial image *P*_0_ of the target vehicle to extract the center point coordinate, preparing for determining the vehicle heading direction.
(4) The RSU sends forward command to the target vehicle through the UDP. After the OBU of the target vehicle receives the command, it sends the command to the ECU to control the vehicle forward.
(5) The RSU tracks the target vehicle in real time, and performs a preliminary pose calculation for the real-time image *P_t_* to obtain the center point coordinate.
(6) The vehicle heading direction is judged according to the moving direction of the center point coordinate of the initial image *P*_0_ and the center point coordinate of the real-time image *P_i_* after the vehicle advances.
(7) The small movement distance between the two front and rear images may not be enough to determine the vehicle heading direction due to the high frame rate of the RSU camera. If the vehicle heading direction cannot be judged, return (5). otherwise, continue to track the vehicle in real time.
(8) The RSU sends the pose information of the vehicle to the OBU in real time to complete the whole process of pose detection.

### 3.1. Data Interaction Based on the UDP

In this paper, data sharing between the RSU and OBU is realized through the UDP. The UDP has low resource consumption and fast processing speed, and provides connectionless datagram services to terminal devices. To enable real-time information transmission between RSU and OBU for transmitting small data volumes such as vehicle position and heading angle information, this paper employs UDP for data transmission. Firstly, the OBU transmits its own vehicle position information to the RSU through UDP for the RSU to locate the target vehicle for vehicle pose detection. Secondly, after the RSU locates the target vehicle, it uses UDP to send a forward command to the target vehicle. While sending the command, the RSU captures real-time images of the target vehicle to determine the vehicle heading direction. Finally, during the vehicle pose detection phase, the RSU continuously detects the vehicle in real time, calculates the pose, and then transmits the pose information to the OBU.

**Figure 2:**
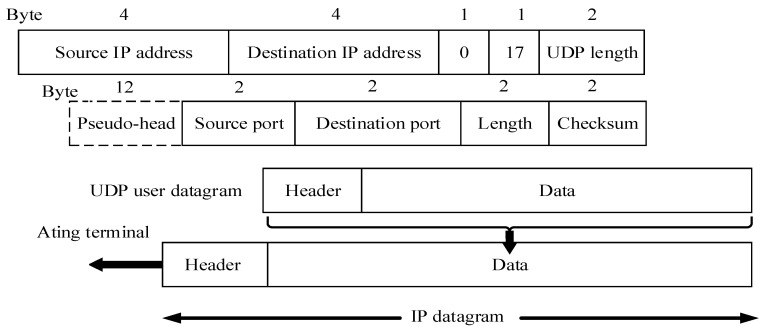
UDP message format.

Before transferring data, the two communicating parties using the UDP do not need to establish a connection, so there is no delay in the connection establishment. With the development of network technology, especially in short-distance transmission areas, UDP data transmit well in real time relative to TCP. Figure 2 shows the format of the UDP message. The UDP message is divided into two parts: UDP message header and UDP data area. The header consists of the source port, the destination port, the message length, and the checksum. When the source host sends the data to the target host, it only needs to send the packet to the IP address and port number of the target host without any pre-connection. If the target host is ready to receive the data, it will receive and process the packet. The application layer protocol will guarantee the reliability of communication. At the same time, because UDP only provides a low level of error control, in the process of data transmission, the transmission delay and the system running memory is small, and the data transmission speed is high, which can be suitable for data transmission in scenarios with high real-time requirements.

**Figure 3:**
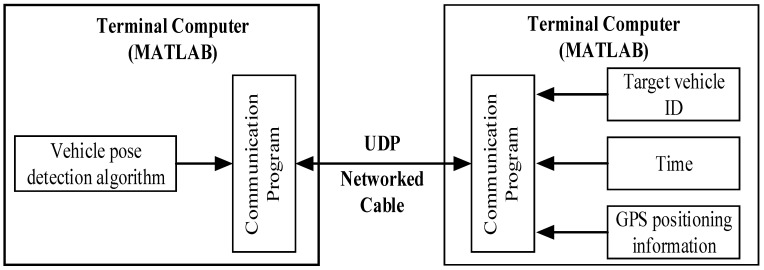
Communication port architecture.

Due to the lack of existing UDP communication programs in MATLAB (MathWorks, Natick, MA, USA), the programs are written on the computers of RSU end and OBU end to receive and transmit data. Figure 3 shows the architecture of the communication port. The vehicle pose detection algorithm is provided on the RSU terminal computer, and the OBU terminal computer provides the target vehicle ID, time, and GPS positioning information. All the information is transmitted through the communication port built by the UDP.

### 3.2. Vehicle Pose Detection

#### 3.2.1. Vehicle Rectangular Fitting

**Figure 4:**
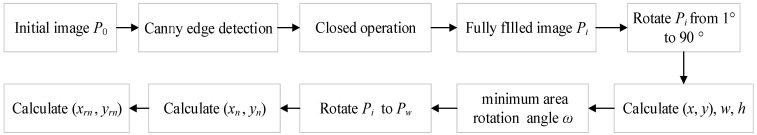
Flow chart of the target vehicle rectangular fitting.

**Figure 5:**
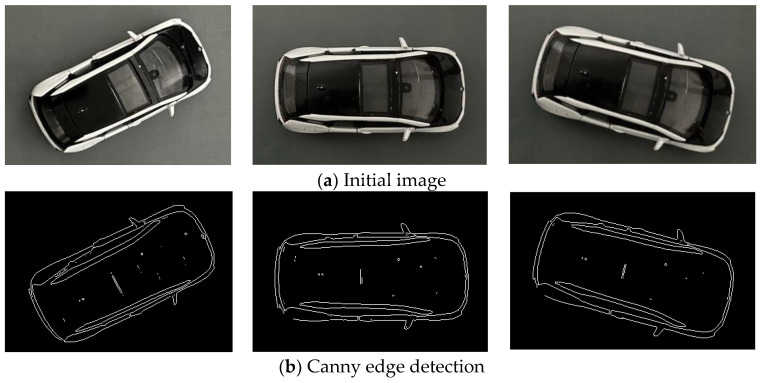

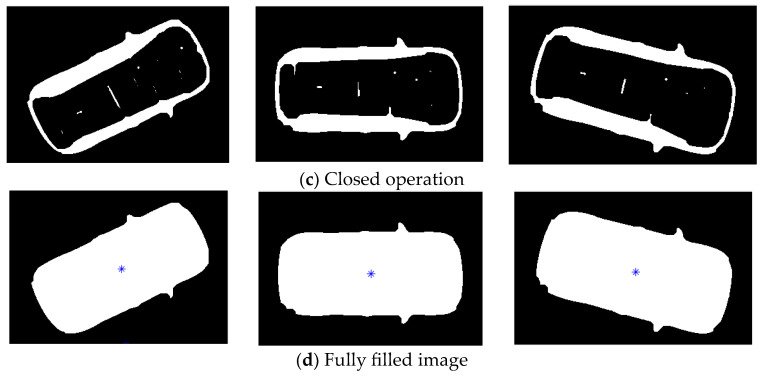
Drawing effect of the target vehicle extraction (the **left**, **middle**, and **right** images show the image processing effects in different directions).

**Figure 6:**
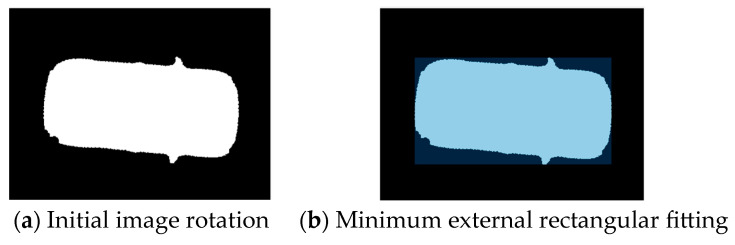
Rectangle outline extraction of the target vehicle.

**Figure 7:**
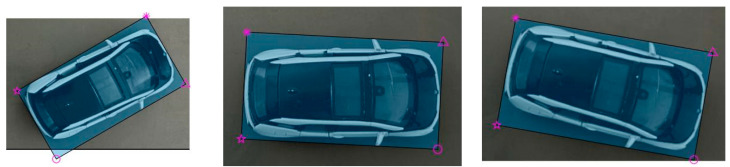
Rectangular fitting of the target vehicle (the **left**, **middle**, and **right** images show the final effects in different directions).

In the top view, the shape of the vehicle is basically rectangular, so the external rectangle of its outline can accurately reflect the pose of the vehicle. Seeing the vehicle as a rectangle can simplify the process of pose estimation and reduce the calculation complexity. The heading angle and position of the vehicle are estimated more accurately by the boundary and position information of the rectangle. Moreover, the rectangular model is suitable for different types of vehicles and can provide stable fitting results at a variety of perspectives and distances. Figure 4 shows the flow chart of the target vehicle rectangular fitting. Figure 5 shows drawing effect of the target vehicle extraction. Figure 6 shows rectangle outline extraction of the target vehicle. Figure 7 shows rectangular fitting of the target vehicle.

(1) The initial image *P*_0_ is used for canny edge detection [22,23,24]. Firstly, Gaussian blur is performed to reduce the effect of noise in the images on edge detection. Secondly, the gradient of the image is calculated using the Sobel operator. Thirdly, the image is processed by non-maximum suppression and refined in the gradient direction to make the edge more refined and accurate. Then, double threshold detection is performed to divide the edge pixels of the image into three categories: strong edge, weak edge, and non-edge. By setting two thresholds, the pixels in the gradient image are divided into high-threshold and low-threshold pixels, where the high-threshold pixels are strong edges and the low-threshold pixels are weak edges. Finally, the weak edge points are connected to connect them with the strong edge points to form the complete edges.
(2) The image after edge detection is processed by closed operation. The image is corroded to eliminate small holes and small connection areas in the image, making the target more compact. After the corrosion operation, the image is expanded to increase the target in the image and fill the holes in the target to make the target more complete.
(3) Fully fill in the image. The image after closed operation is completely filled, the convex packets and areas of the image are calculated, the area of the convex packets is calculated again, the target with the largest area is retained, the centroid is extracted for further target analysis and processing, the other targets are deleted, and finally, the filled image *P_i_* of the vehicle is obtained.
(4) Rotate *P_i_*. Rotate *P_i_* at different angles (from 1° to 90°) to find the minimum external rectangle.
(5) The bounding box information is calculated for each rotated image, including the upper left coordinate (*x*, *y*), width (*w*), and height (*h*) of the bounding box.
(6) Calculate the minimum area angle *ω*. The area of each rotated image is calculated based on the width and height of the bounding box to find the rotation angle that makes the area of the external rectangle minimum.
(7) The image *P_i_* is rotated according to the optimal angle found to obtain the final rotated image *P_w_*.
(8) The coordinates of the four corner points (*x_n_*, *y_n_*) (*n* = 1, 2, 3, 4) after rotation are calculated to complete the fitting process of the image minimum external rectangle.
(9) The coordinates of the four corner points (*x_rn_*, *y_rn_*) (*n* = 1, 2, 3, 4) of the external rectangle in the initial image *P*_0_ are calculated based on the rotation angle *ω*, completing the final process of rectangular fitting.

#### 3.2.2. Vehicle Pose Calculation Model

**Figure 8:**
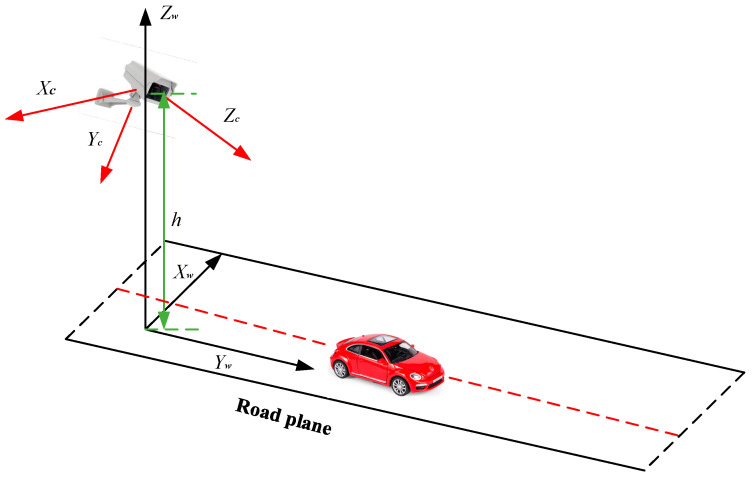
Schematic diagram of the road camera (RSU camera) model.

The roadside equipment end mainly detects the environment within its visual range through the camera loaded on the road experiment unit. The installation height and the pose angle of the camera can be set in advance. Figure 8 is the RSU camera model, which shows the position relationship between the RSU camera and the road plane. *X_c_*, *Y_c_*, and *Z_c_* are the axes of the camera coordinate system. *X_w_*, *Y_w_*, and *Z_w_* are the axes of the world coordinate system. The installation height of the RSU camera is *h*.

**Figure 9:**
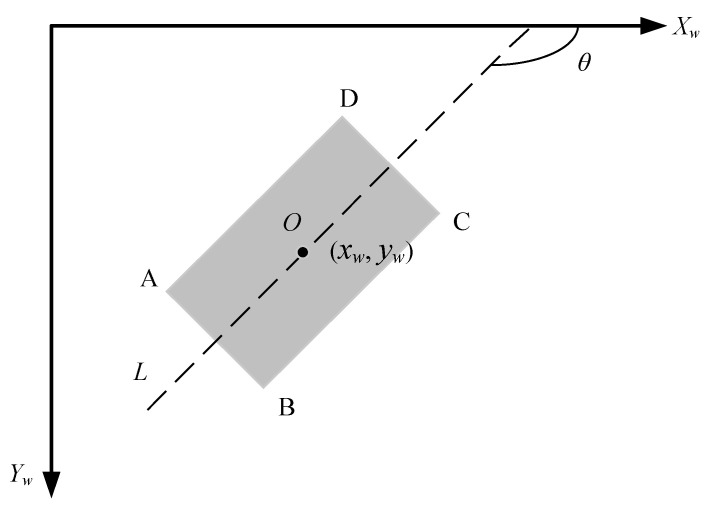
Schematic diagram of the pose calculation.

The vehicle heading angle refers to the angle between the center speed of the vehicle and the horizontal axis under the world coordinate system. At low speeds, the movement of the vehicle in the vertical direction is usually negligible, so the center velocity direction of the vehicle is consistent with the longitudinal central axis of the vehicle. After obtaining the vehicle rectangular fitting box, the coordinates of the four corner points in the image coordinate system can be obtained: A_1_ (*x_c_*_1_, *y_c_*_1_), B_1_ (*x_c_*_2_, *y_c_*_2_), C_1_ (*x_c_*_3_, *y_c_*_3_), and D_1_ (*x_c_*_4_, *y_c_*_4_). Converting the coordinates of the four corner points into the world coordinate system, A (*x_w_*_1_, *y_w_*_1_), B (*x_w_*_2_, *y_w_*_2_), C (*x_w_*_3_, *y_w_*_3_), and D (*x_w_*_4_, *y_w_*_4_) are obtained. Figure 9 shows the position of the four angle points of the vehicle in the world coordinate system. Through the coordinates of the four angle points, the angle between the central axis *L* and the horizontal coordinate axis, which is the calculated heading angle *θ* and the vehicle center point *O*, taken as the positioning point, can be obtained.

(1) $$\tan\theta =\frac{y_{w4}+y_{w3}-y_{w2}-y_{w1}}{x_{w4}+x_{w3}-x_{w2}-x_{w1}}$$

(2) $$\theta =\arctan\frac{y_{w4}+y_{w3}-y_{w2}-y_{w1}}{x_{w4}+x_{w3}-x_{w2}-x_{w1}}$$

(3) $$\left(x_{w},y_{w}\right)=\left(\frac{x_{w1}+x_{w2}+x_{w3}+x_{w4}}{4},\frac{y_{w1}+y_{w2}+y_{w3}+y_{w4}}{4}\right)$$

**Figure 10:**
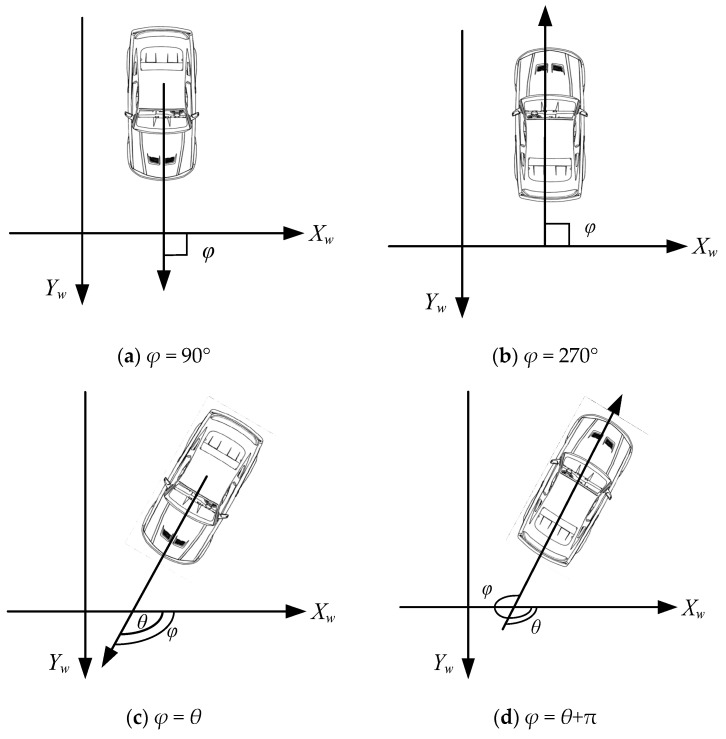
The schematic diagram of the conversion relationship between actual heading angle and calculated heading angle. (**a**) The schematic diagram when heading angle is 90°; (**b**) the schematic diagram when heading angle is 270°; (**c**,**d**) the schematic diagram of heading angle calculation in other cases.

In practice, there is a difference between the calculated heading angle and the actual heading angle, so it is stipulated that when the vehicle heading orientation is consistent with the front half axis of the *X_w_* in the world coordinate system, the heading angle is 0°, and the clockwise heading angle is positive. Then, the relationship between calculated heading angle *θ* and actual heading angle *φ* is shown in Figure 10.

#### 3.2.3. Vehicle Heading Direction Detection Model

**Figure 11:**
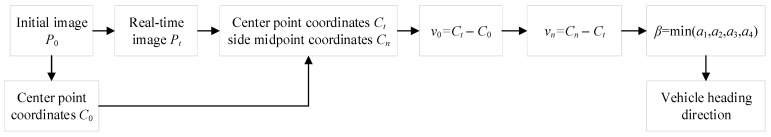
Flow chart of the vehicle heading direction judgment.

**Figure 12:**
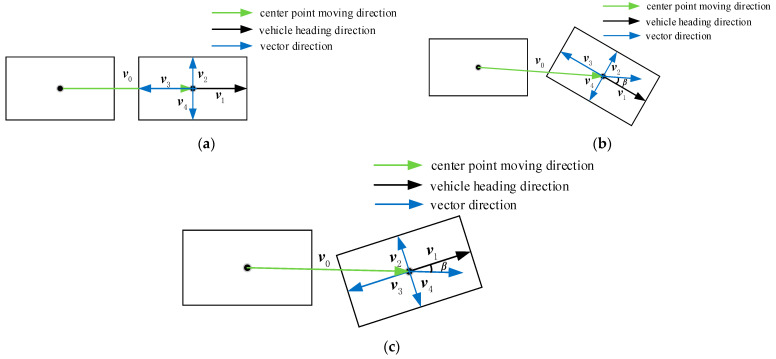
Schematic diagram of the vehicle heading direction judgment. (**a**), (**b**) and (**c**) represent the judgment of the heading direction in different situations, respectively.

The judgment of the vehicle heading direction is an important process of the vehicle heading angle detection. In this paper, the forward command is sent to the vehicle through communication to control the vehicle forward and the vehicle heading direction is judged by the moving direction of the vehicle center point. When the vehicle takes the rectangular fitting, the movement direction of the center point of the vehicle may not be on the same straight line, that is, there is an angle. In this case, the movement direction of the center point of the vehicle cannot be simply used as the vehicle heading direction, and the vehicle heading direction needs to be accurately judged to calculate the pose. Figure 11 shows the specific steps. Figure 12 shows the schematic diagram of the vehicle heading direction judgment.

(1) Calculate the initial image pose. Obtain the initial image *P*_0_, conduct the preliminary pose calculation for it, and obtain the center point coordinate *C*_0_.
(2) Calculate the real-time image pose. Conduct the preliminary pose calculation for the real-time image *P_t_* to obtain the center point coordinate *C_t_*, and the four side midpoint coordinates *C*_1_, *C*_2_, *C*_3_, *C*_4_.
(3) Calculate the displacement vector *v*_0_ and the side midpoint vector *v_n_*. The center point displacement vector $v_{0}=C_{t}-C_{0}$. Calculate the side midpoint vectors *v*_1_, *v*_2_, *v*_3_, *v*_4_ from the center point of the vehicle *C_t_* obtained through the real-time bounding box to the midpoints of the four sides of the rectangle *C*_1_, *C*_2_, *C*_3_, *C*_4_.
  (4) $$v_{n}=C_{n}-C_{t}\;(n=1,2,3,4)$$
(4) Calculate the minimum clip angle *β*. Calculate the angles *a*_1_, *a*_2_, *a*_3_, *a*_4_ between the displacement vector *v*_0_ and the side midpoint vectors *v*_1_, *v*_2_, *v*_3_, *v*_4_.
  (5) $$a_{n}=\arccos\left(\frac{v_{0}\cdot v_{n}}{\left\|v_{0}\right\|\left\|v_{n}\right\|}\right)\;(n=1,2,3,4)$$
(5) The minimum vector clip angle *β* is obtained:
  (6) $$\beta =\min\left(a_{1},a_{2},a_{3},a_{4}\right)$$
(6) Determine the vehicle heading direction. The vector direction corresponding to the minimum clip angle *β* is the vehicle heading direction.

## 4. Experiment and Results Analysis

### 4.1. Object Detection

#### 4.1.1. Dataset Training

**Figure 13:**
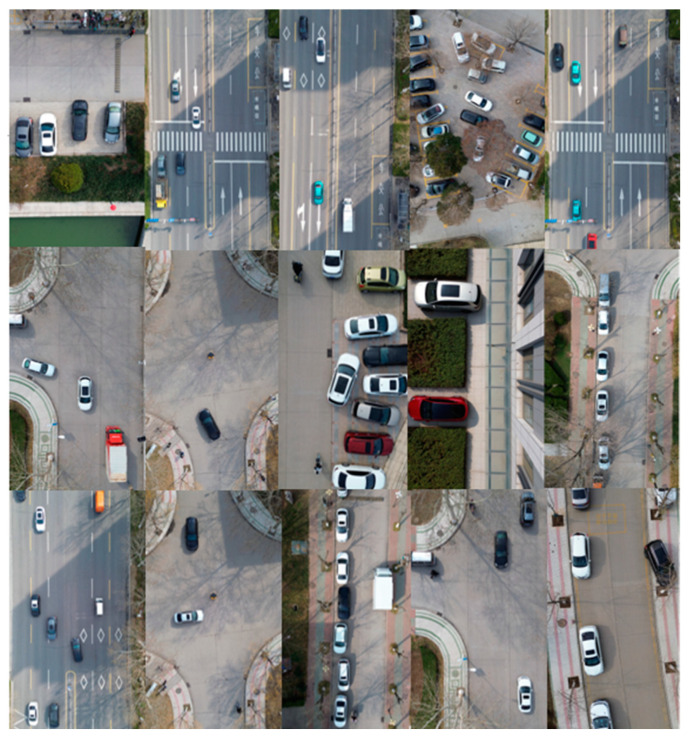
Part of the dataset images.

The vehicle pose detection method proposed in this paper calculates the vehicle pose by vehicle rectangular fitting. In order to identify the target vehicles in the traffic scenes, it is necessary to detect the vehicles from the overhead perspective. The current vehicle detection algorithms cannot meet the practical application requirements of the vehicle detection from the overhead perspective, so the dataset of the vehicles from the overhead perspective is collected and trained to realize the detection of the target vehicles. The dataset used in this paper consists of 3190 vehicle images captured by UAV, including images with occlusions and scale variations. The training set comprises 1724 images, the validation set comprises 175 images, and the test set comprises 1291 images. Part of the dataset images is shown in Figure 13.

**Figure 14:**
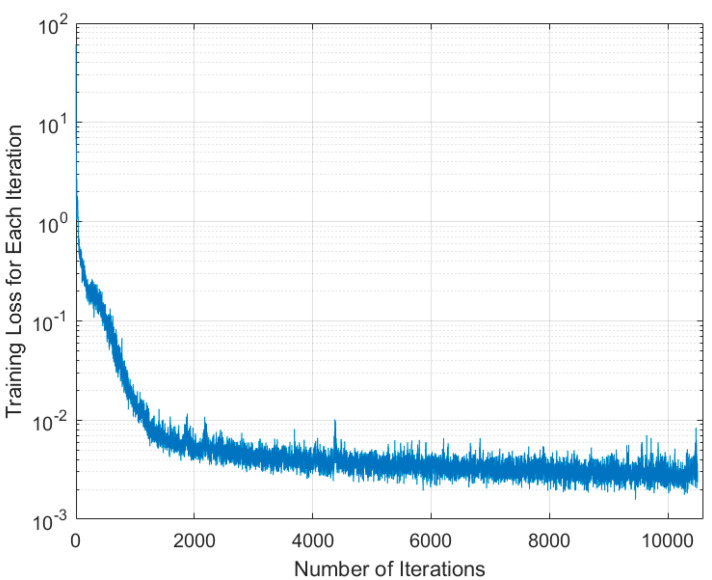
Training loss for each iteration.

During the training process, the model was optimized using the stochastic gradient descent optimizer. The initial learning rate is 0.01, the MiniBatchSize is set to 20, the MaxEpochs is set to 300, and the input resolution of the image is 1530 × 2720 [25,26,27]. Figure 14 shows the training loss for each iteration.

#### 4.1.2. Training Accuracy

In order to evaluate the training effect more accurately, the evaluation indexes such as detection accuracy, true accuracy, and overall accuracy were used to evaluate the model performance. The calculation method is shown in Equations (7)–(9):

(7) $$OT=\frac{FN+FP}{TP+FP+TN+FN}$$

(8) $$DT=\frac{FP}{TP+FP}$$

(9) $$RT=\frac{FP}{TN+FP}$$

*TP* is the number of samples detected as vehicles and actually vehicles. *FP* is the number of samples detected as vehicles but not actually vehicles. *TN* is the number of samples detected as not vehicles but actually vehicles and *FN* is the number of samples detected as not vehicles and actually not vehicles. According to the detection results, the confusion matrix [28,29,30,31,32] was obtained as shown in Table 1.

It can be seen from Table 2 that the effect of the training for vehicles detection is remarkable, which can meet the needs of target vehicle identification for pose detection.

### 4.2. Pose Detection Experiment of the Real Vehicle Scenarios

**Figure 15:**
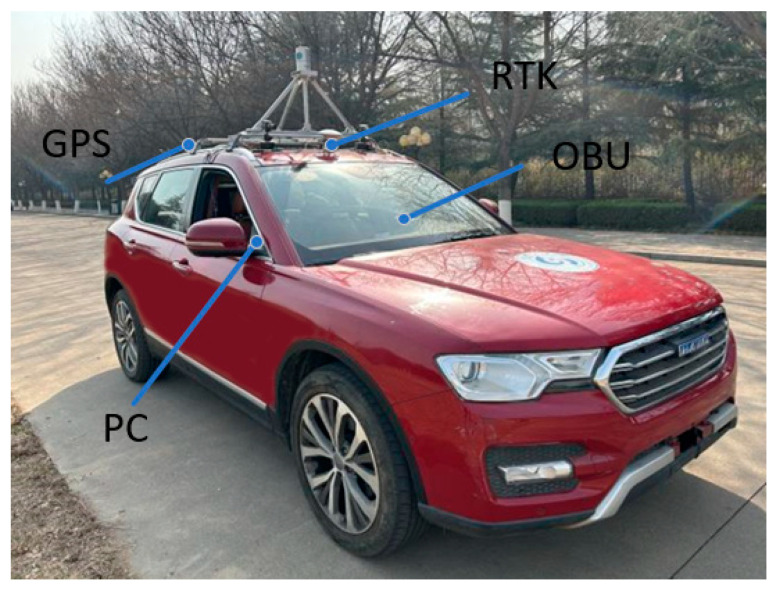
Experimental vehicle equipment.

**Figure 16:**
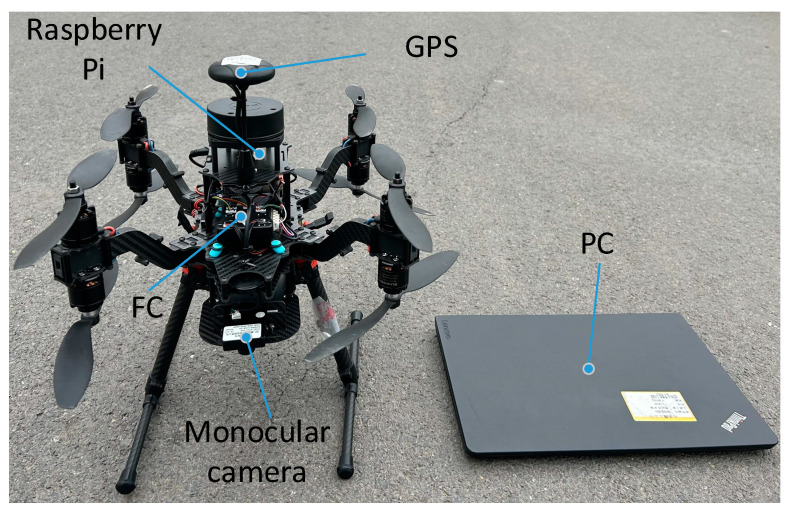
RSU equipment.

**Figure 17:**
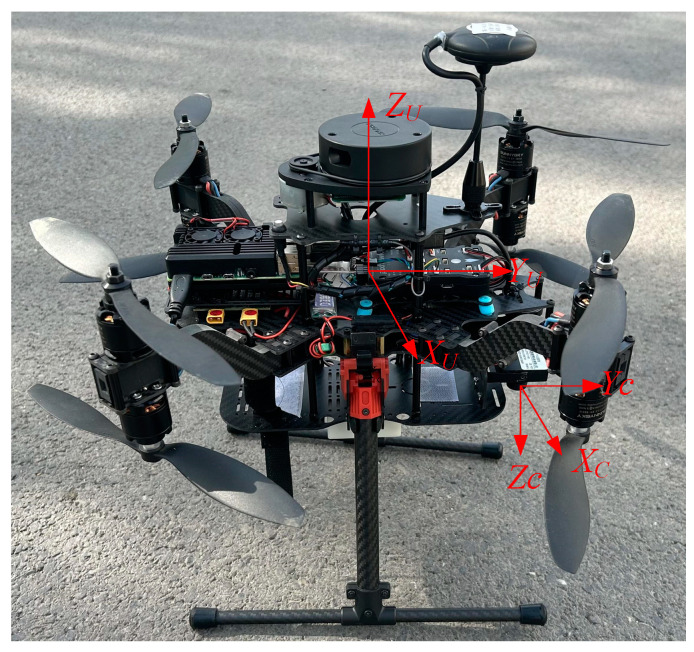
The relative position of the UAV and the camera.

The experiment was operated as follows. The UAV and the terminal computer form the RSU equipment, in which the UAV camera is the RSU camera, and the terminal computer realizes the image and data processing and communicates with the vehicle OBU. To enable the UAV to simulate the RSU as much as possible, the experiment was conducted in a windless environment. The UAV flies to a certain height and then hovers. The UAV camera should be kept parallel to the ground, namely, the pitch angle is 0°. When the vehicle enters the RSU perception area, the OBU sends the on-board GPS position information to the RSU through UDP. After the RSU accepts the information, the vehicle is obtained and the initial image obtained by the RSU is sent to the RSU through Raspberry PI for processing. The RSU computer first conducts the target detection. By matching the result of the target detection and the GPS information, the detection target is considered as the actual target vehicle. The preliminary pose calculation of the target vehicle was performed to obtain the coordinate of the vehicle center point. At this point, the RSU sends a forward command to the vehicle, and the OBU receives the command and forwards it to the ECU to control the vehicle to move forward. The RSU tracks the vehicle and completes the preliminary pose calculation of the vehicle in real time to obtain the coordinate of the vehicle center point. By comparing the initial center point coordinate of the vehicle with the real-time center point coordinate after moving forward, the vehicle heading direction can be determined. Subsequently, the RSU continues to track the vehicle, detect its pose, obtain the center coordinates of the vehicle and the heading angle information, and transmit the information to the OBU, completing the entire process of vehicle pose detection. To verify the effectiveness of the vehicle pose detection method based on the RSU under different driving modes, the experiments were conducted in both straight-line and turning driving states. Table 3 shows UAV parameters, Figure 15 shows experimental vehicle equipment, Figure 16 shows RSU equipment and Figure 17 shows the relative position of the UAV and the camera.

**Figure 18:**
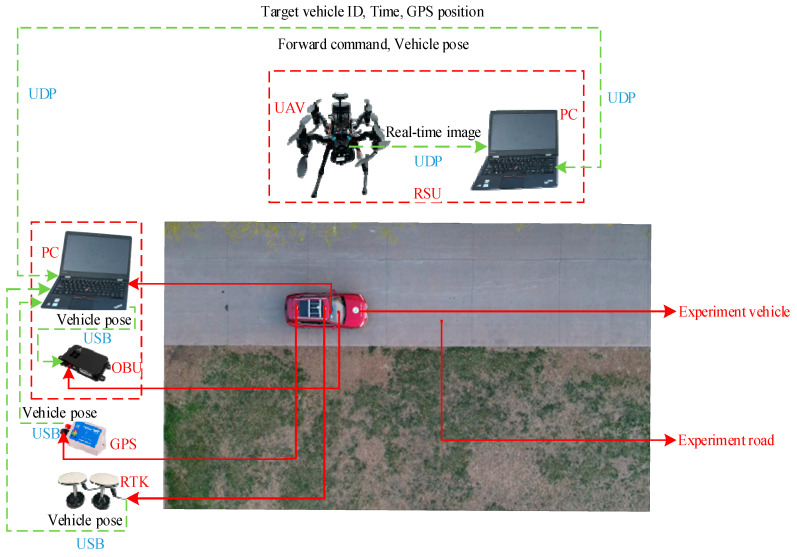
System equipment.

**Figure 19:**
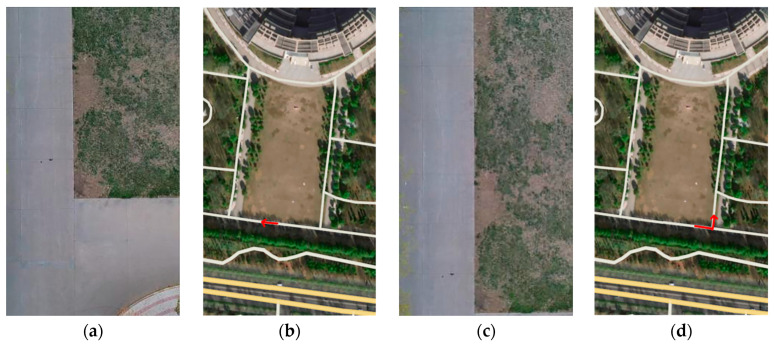
Experiment scenarios. (**a**) shows the actual experiment route of the straight-line scenario; (**b**) shows the map identification route of the straight-line scenario; (**c**) shows the actual experiment route of the turning scenario and (**d**) shows the map identification route of the turning scenario.

**Figure 20:**
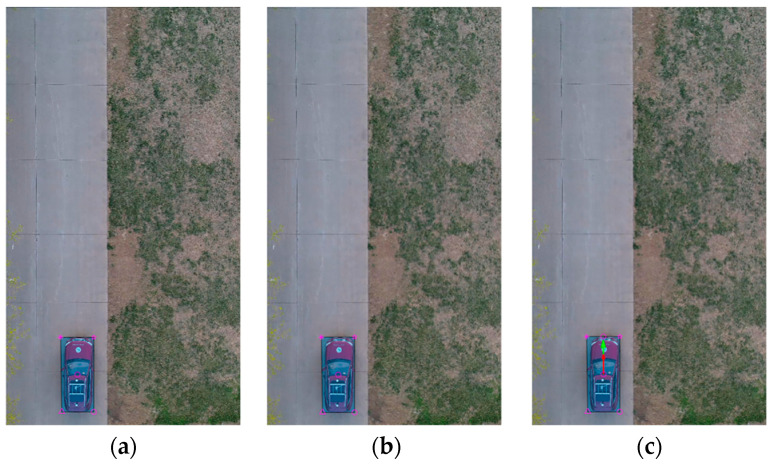

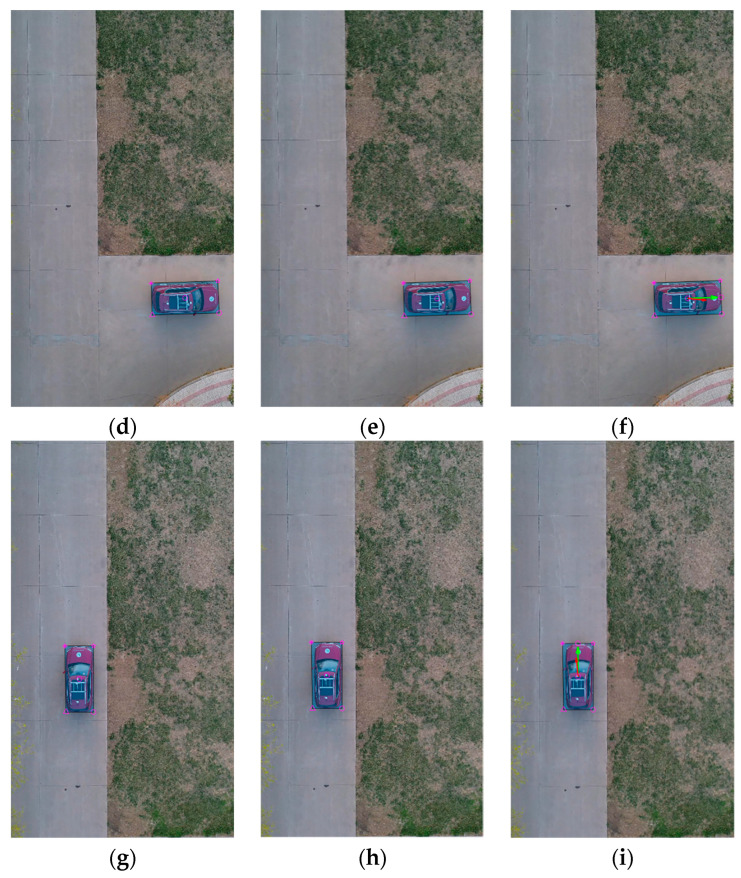
The vehicle heading direction detection. (**a**–**c**) show the detection effect of the head with no deviation between the moving direction of the center point and the heading direction; (**d**–**i**) are the detection effect of the heading direction with different deviations.

**Figure 21:**
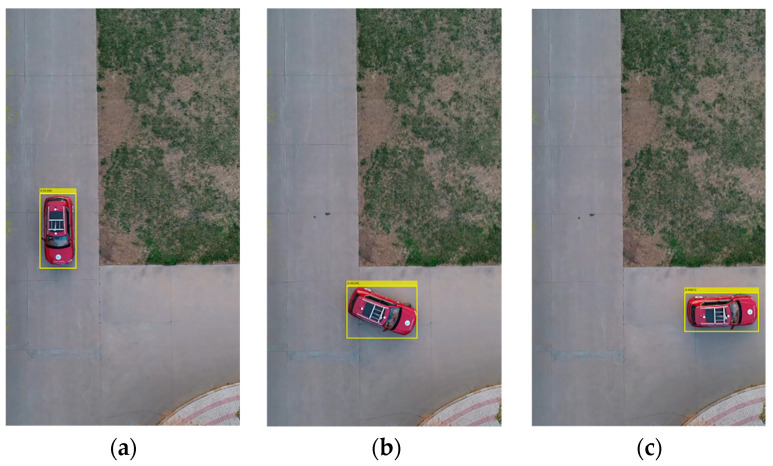
Target vehicle detection. (**a**–**c**) represent the target vehicle detection results at different times, respectively.

**Figure 22:**
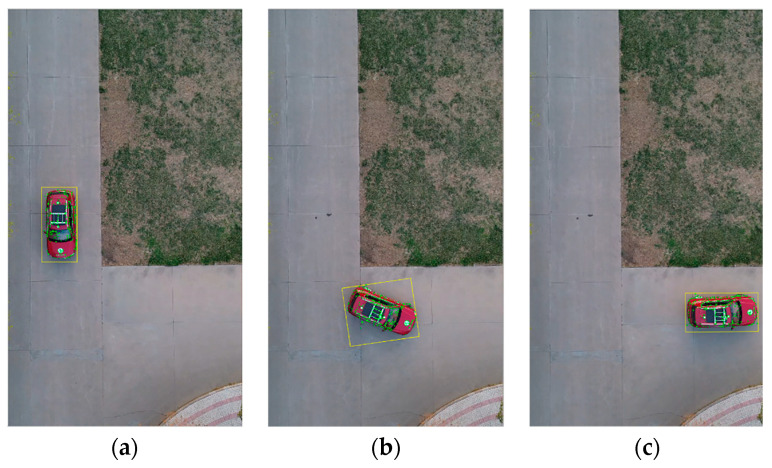
Target vehicle tracking. (**a**–**c**) represent the target vehicle tracking results at different times, respectively.

**Figure 23:**
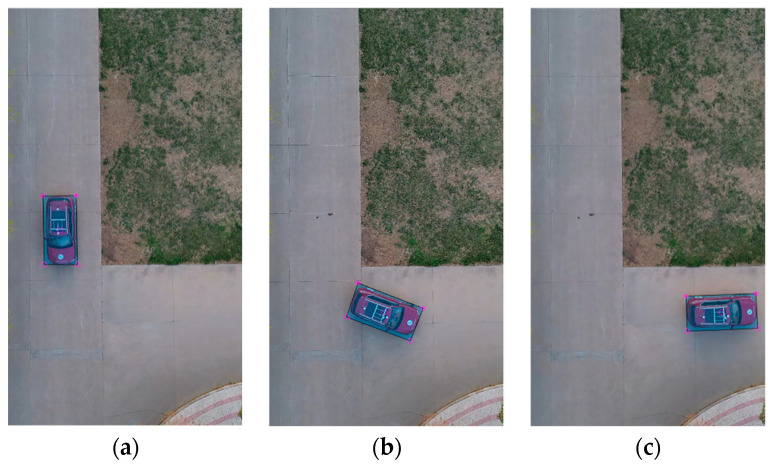
Target vehicle pose calculation. (**a**–**c**) represent the target vehicle pose calculation results at different times, respectively.

**Figure 24:**
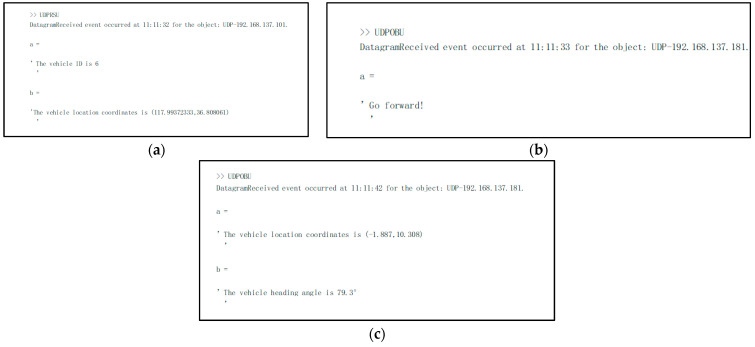
The communication results diagram. (**a**) The vehicle ID and GPS information received by MATLAB in the RSU; (**b**,**c**) the information received by MATLAB in the OBU, which includes the forward command information and pose information, respectively.

The vehicle heading direction detection experiments were conducted. Figure 18 shows the system equipment (the red represents device name and installation location, the blue represents communication mode, and the black represents data transmission content). The experiment scenarios are shown in Figure 19 (the red arrow indicates the driving direction of the vehicle). Figure 19a shows the actual experiment route of the straight-line scenario (where the green arrow indicates the heading direction and the red arrow is the moving direction of the center point); Figure 19b shows the map identification route of the straight-line scenario; Figure 19c shows the actual experiment route of the turning scenario; Figure 19d shows the map identification route of the turning scenario. The experiment results are shown in Figure 20 (the green arrow indicates the heading direction and the red arrow is the moving direction of the center point). Figure 20a–c show the detection effect of the head with no deviation between the moving direction of the center point and the heading direction; Figure 20d–i are the detection effect of the heading direction with different deviations. As can be seen from the figures, the heading direction detection method has a significant effect. Figure 21, Figure 22 and Figure 23 shows the results of target vehicle detection, target vehicle tracking and target vehicle pose calculation, respectively. Figure 24 shows the communication results.

### 4.3. Detection Speed Analysis

The average time used by the vehicle pose detection method based on the RSU and the GPS method was compared to obtain the pose detection data, and the detection speed of the two methods was compared. The detection time is shown in Table 4.

Table 4 shows that the vehicle pose detection method based on the RSU takes less time and detects faster when obtaining the pose detection data than the GPS-based method. This is because this method obtains many image data, and the pose solution process is simple, while data obtained by GPS have a low frequency, and there is a delay. Therefore, the detection speed of the vehicle pose detection method based on the RSU method is fast.

### 4.4. Detection Accuracy Analysis

**Figure 25:**
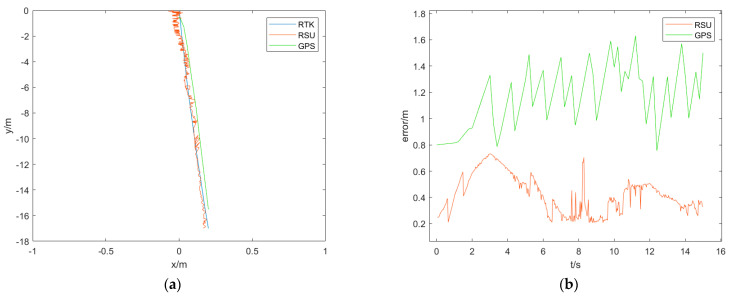

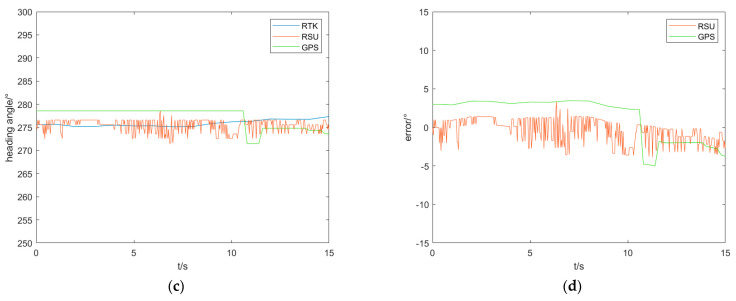
Results of the straight-line scenario. (**a**) The absolute trajectory comparison result of the straight-line scenario; (**b**) the absolute trajectory error comparison result of the straight-line scenario; (**c**) the heading angle comparison result of the straight-line scenario; (**d**) the heading angle error comparison result of the straight-line scenario.

**Figure 26:**
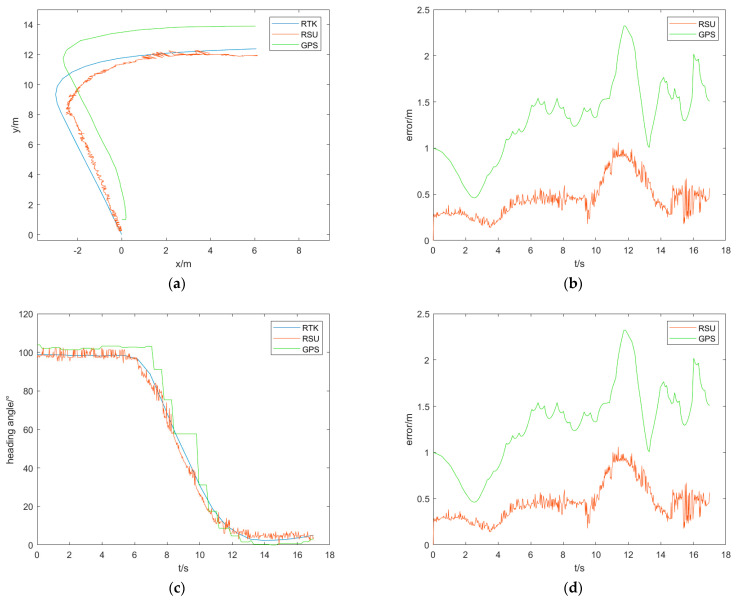
Results of the turning scenario. (**a**) The absolute trajectory comparison result of the turning scenario; (**b**) the absolute trajectory error comparison result of the turning scenario; (**c**) the heading angle comparison result of the turning scenario; (**d**) the heading angle error comparison result of the turning scenario.

The trajectory and heading angle plots obtained from the vehicle pose detection method based on the RSU and the GPS-based method are shown in the figure below. Figure 25 and Figure 26 shows the results of the straight-line scenario and the turning scenario, respectively. Due to the different data acquisition frequencies of this method, GPS, and RTK (which has a lower frequency), interpolation was performed on the RTK data to facilitate error analysis. As the data trend obtained in this process from RTK is relatively regular, linear interpolation is used to interpolate the data according to the frequencies of RSU and GPS to obtain the interpolated results. A comparison is then made between the interpolated results and the detection results from RSU and GPS, generating error plots. A detailed analysis of the errors is presented in the table below. The columns in the table represent max for maximum error, min for minimum error, mean for average error, std for standard deviation, and rmse for root mean square error. All values are in meters.

From the Table 5, it can be seen that whether in the straight-line scenario or the turning scenario, the performance of the vehicle pose detection method based on the RSU is overall better than the GPS-based method. This is because the vehicle pose detection method based on the RSU has strong adaptability to the environment and a faster detection speed, enabling it to capture the changes in the vehicle pose during driving in a timely manner. On the other hand, GPS has positioning delays, making it unable to reflect real-time changes in the vehicle pose. Additionally, during turning maneuvers where the environment changes significantly, GPS accuracy is affected by environmental factors, leading to decreased precision. The method presented in this paper exhibits some jitter phenomenon, which is attributed to the unstable process of rectangular fitting, particularly in the fitting of the rearview mirror. The instability occurs during the rectangular fitting process; where both rearview mirrors may be fitted, only one rearview mirror may be fitted, or no rearview mirror may be fitted, thereby affecting the precise detection of trajectory points. Furthermore, during the target detection process, parts of the environment that do not belong to the vehicle are also detected, leading to the extraction of other feature points during pose detection and impacting the final detection results. In summary, the vehicle pose detection method based on the RSU offers a higher accuracy and faster detection speed, making it suitable for real-world vehicle scenarios.

## 5. Conclusions

This paper presents a pose detection method based on the RSU method. In the process of vehicle pose calculation, the paper analyzes the pose information of vehicles with different heading orientations to avoid pose misjudgments caused by vehicle orientation. Comparison experiments were conducted outdoors between the GPS-based pose detection method and the method in this paper. The experimental results of the position and heading angle were compared using mean, max, min, std, and rmse as evaluation metrics. The results indicate that, due to the simplicity and stability of the detection method in this paper, it has a high accuracy. The detection speed of the GPS-based pose detection method was compared with this method, and the processing speed of this method is faster due to the high frame rate of the RSU camera.

The method presented in this paper has the advantages of high accuracy and fast processing speed, and is suitable for various vehicle scenarios such as parking lots, areas with dense and high buildings, and cargo yards. This paper is innovative in the pose detection field, using the method based on the RSU to realize the accurate detection of the vehicle pose. However, the security of the data transmission process in this paper is still insufficient. The subsequent research will focus on the security direction of the data transmission process, and ensure the security of the data transmission process by strengthening the encryption algorithm, adopting two-factor authentication, and establishing the secure data channels to confront the increasingly severe cyber security threats. The problems of increased transmission time brought by these measures will also be considered. At the same time, regarding the increased communication delay that may arise from a single RSU serving multiple OBUs, multi-channel and time allocation, priority and quality of service management, and channel management and resource allocation will be considered to improve the performance of the method proposed in the paper.

## Figures and Tables

**Table 1:** The confusion matrix of the detection results.

		True	Actual	None or False
Detection result	True	1203		25
	None or false	29		34

**Table 2:** Detection accuracy.

	OT	DT	RT
YOLOv2 detection	0.958	0.980	0.976

**Table 3:** Table of UAV parameters.

CQ 360 UAV Development Platform			
Version	CQ360-X8	Endurance time	25 min
Flying height	21 m	Pitch angle	0°
Yaw angle	5.32°	Roll angle	0.29°
Battery	4 s (16.8 V)	Remote control distance	1500–2000 m
Dimension	300 × 300 × 350 mm	Wind resistance level	Level 3–4

**Table 4:** Comparison of the average detection time.

Methods	RSU	GPS
Detection time/s	0.063	0.235

**Table 5:** Error analysis.

	Methods	Max	Min	Mean	Std	Rmse
Straight-line track (location) error	RSU	0.732	0.213	0.422	0.143	0.446
	GPS	1.631	0.75	1.163	0.226	1.184
Straight-line heading angle error	RSU	3.511	0.014	1.313	1.038	1.426
	GPS	5.015	1.816	3.100	0.679	3.085
Turning track (position) error	RSU	1.063	0.242	0.455	0.201	0.498
	GPS	2.323	0.495	1.273	0.417	1.340
Turning heading angle error	RSU	5.648	0.037	2.343	1.642	2.060
	GPS	23.044	0.667	4.807	4.142	6.345

## Data Availability

Data are contained within the article.
